# Optimizing gestational diabetes diagnostic criteria to predict adverse perinatal outcomes in the United Arab Emirates: The Mutaba’ah Study

**DOI:** 10.3389/fendo.2025.1641326

**Published:** 2025-10-10

**Authors:** Maryam M. Bashir, Luai A. Ahmed, Rami H. Al-Rifai, Iffat Elbarazi, Tom Loney, Bachar Afandi, Juma M. Alkaabi, Fatma Al-Maskari

**Affiliations:** ^1^ Institute of Public Health, College of Medicine and Health Sciences, United Arab Emirates University, Al Ain, Abu Dhabi, United Arab Emirates; ^2^ Zayed Centre for Health Sciences, United Arab Emirates University, Al Ain, Abu Dhabi, United Arab Emirates; ^3^ College of Medicine, Mohammed Bin Rashid University of Medicine and Health Sciences, Dubai Health, Dubai, United Arab Emirates; ^4^ Endocrinology Division, Tawam Hospital, SEHA, Al Ain, United Arab Emirates; ^5^ Department of Internal Medicine, College of Medicine and Health Sciences, United Arab Emirates University, Al Ain, Abu Dhabi, United Arab Emirates

**Keywords:** gestational diabetes mellitus (GDM), diabetes, diagnostic criteria, IADPSG, perinatal outcomes, oral glucose tolerance test (OGTT), risk stratification, United Arab Emirates

## Abstract

**Background:**

Gestational diabetes mellitus (GDM) affects 25% of pregnancies in the United Arab Emirates (UAE), and there is a need for evidence-based diagnostic criteria. This study aimed to determine the optimal diagnostic criteria for GDM in the Emirati population based on predicting adverse perinatal outcomes.

**Methods:**

A total of 2,449 eligible pregnancies from “The Mutaba’ah Study” birth cohort were screened using OGTT between 24 and 32 weeks from May 2017 to March 2021. We compared the prediction of adverse perinatal outcomes [Large for Gestational Age (LGA) and Composite Outcome] risk by four GDM diagnostic criteria (IADPSG, NICE2015, WHO1999, and ADIPS1998) using adjusted regression models. We then developed a new GDM diagnostic threshold for this population (using an aOR of 1.75 recommended by the IADPSG consensus panel). The new criteria was validated and compared with other criteria using risk analyses, c-statistic (AUC), integrated discrimination improvement (IDI), and net reclassification improvement (NRI).

**Results:**

Of the four criteria assessed, IADPSG was the best predictor for large for gestational age (LGA) *(aOR 1·77, 95%CI 1·36-2·29)* and composite outcome *(aOR 1·49, 95%CI 1·19-1·86)*. The newly developed criteria showed even stronger associations than the IADPSG [LGA *(aOR 1·93, 95% CI 1·48-2·53)*; Composite Outcome *(aOR 1·62, 95% CI 1·28-2·05)*]. The new criteria model had good discrimination properties for LGA prediction *(AUC 0·78; 95%CI 0·68-0·88)* and composite outcome prediction *(AUC 0.73; 95%CI 0.57-0.83)*. The new criteria model also correctly reclassifies 49.4% of patients based on LGA risk *[NRI; 0·494 (p=0·043)]*, whereas the IADPSG did not significantly reclassify these patients *[NRI; 0·202 (p=0·409)]*. For composite outcome prediction, the NRIs for both models were not statistically significant. The new criteria model also improved the discrimination slope (IDI) for LGA prediction by 42.2%, whereas IADPSG improved it by only 9.0%. For the composite outcome prediction, the new criteria model improved by 5.0% vs. IADPSG by 1.3%.

**Conclusions:**

Following the development of a new threshold, the GDM diagnostic criteria defined in this study predicted adverse perinatal outcomes better and demonstrated more optimal clinical utility compared with the existing criteria in this population; hence, adopting it could minimize the burden of GDM adverse perinatal outcomes.

## 1 Introduction

Gestational diabetes mellitus (GDM) is a crucial public health problem with both short- and long-term consequences (1). It affects one in seven live births globally (2) and one in four in the United Arab Emirates (UAE) (3). If undiagnosed and untreated, it is associated with multiple adverse perinatal outcomes (4), future risk of type 2 diabetes (5, 6), childhood adiposity (7), childhood insulin resistance (8), and high economic burden (9, 10).

Although using evidence-based GDM diagnostic criteria is paramount in a population to effectively identify, manage cases, and prevent adverse outcomes (11), the controversy on accurate screening and diagnosing GDM has been ongoing for approximately six decades, and the search for a global consensus guideline has remained elusive (12, 13). There are many international guidelines on GDM diagnostic criteria, and only a few are evidence-based. The International Association of Diabetes and Pregnancy Study Group (IADPSG) criteria (14) is the only GDM diagnostic criteria originally developed based on the risk of developing adverse perinatal outcomes and has been ratified by many international organizations and adopted in many countries, including the UAE (15–17).

The HAPO study, which the IADPSG criteria was based on, was a sizeable multiethnic study; nevertheless, such study is yet to be conducted in the Arab populations (18). A subgroup analysis of the HAPO study data conducted in 2022 showed variations in adverse perinatal outcome predictions by different criteria based on ethnicity (19). The Emirati population of the UAE has one of the highest burdens of GDM worldwide (3, 20).

Several studies have validated using the IADPSG criteria in different populations and compared it with other criteria (21–23). Despite its popularity and use, the IADPSG criteria has still not resolved some issues of utmost concern regarding GDM diagnosis. These include variations in different populations, likely due to resource availability, expertise, burden of GDM adverse perinatal outcomes, and ethnicities (12).

In the UAE, studies have highlighted that different doctors and health facilities are utilizing multiple GDM diagnostic criteria and guidelines (24, 25). Optimal criteria that correctly classify patients based on their risk of developing adverse perinatal outcomes are needed to ensure more optimal management and improved GDM care. This study aimed to compare four GDM diagnostic criteria [IADPSG (14), National Institute for Health and Clinical Excellence (NICE 2015) (26), World Health Organization (WHO 1999) (27), and Australasian Diabetes in Pregnancy Society (ADIPS 1998) (28)] regarding their prediction of adverse perinatal outcomes and to develop the optimal GDM criteria for use among UAE population.

## 2 Materials and methods

### 2.1 Study design and setting

This analysis is based on prospective data collected between 2017 and 2021 in the Mutaba’ah Study, the most extensive multicenter mother and child cohort study in the UAE. It recruits and follows up mother–baby pairs from the Emirati population in Al Ain city, which has the largest proportion of Emiratis in the country. The eligibility criteria for recruitment into the Mutaba’ah study include being an 18-year-old and above pregnant woman from the Emirati population. The study was approved by the United Arab Emirates University Human Research Ethics Committee (ERH-2017-5512) and the Abu Dhabi Health Research and Technology Ethics Committee (DOH/CVDC/2022/72). All participants provided written informed consent.

### 2.2 Study population

For this study, we included the Mutaba’ah study participants with singleton pregnancies screened with a 75-g 2-h oral glucose tolerance test (OGTT) at 24 to 32 weeks between May 2017 and March 2021. Those with at least two OGTT readings were included, and those with preexisting or newly diagnosed diabetes (fasting plasma glucose (FPG) ≥7 mmol/L and/or 2-h OGTT ≥ 11·1 mmol/L) were excluded.

### 2.3 Procedures

Details of the Mutaba’ah study, including the recruitment process, have been published elsewhere (29). Trained research assistants approached eligible pregnant women during their antenatal care visits to any of the participating hospitals. The assistants administer the questionnaires during the first trimester. After delivery, clinical data, including anthropometric measurements, OGTT results, other laboratory results, and data on the index pregnancy, delivery, and outcomes, were extracted from the medical records. The extraction was automated by the IT department of the hospital.

GDM screening is standardized across the three recruiting hospitals as they all follow the Abu Dhabi Department of Health (DOH) guidelines. Standard quality is achieved through internal and external quality control. All pregnant women during routine antenatal care are offered universal GDM screening with 75-g 2-h OGTT at 24 to 28 weeks. Before 24 weeks of gestation, they undergo screening for preexisting diabetes using fasting plasma glucose (FPG) or HbA1C tests, and those positive are being co-managed with endocrinologists. The OGTT procedures are similar across the hospitals. The pregnant women are requested to fast for 8 to 10 h from the night before testing. A venous blood sample is drawn by an expert phlebotomist using standard practice. The women are then given 75 g oral glucose (Trutol, 10 fluid ounces (296 ml) dextrose beverage, Nerl Diagnostic, Rhode Island, USA). After 1 h, a venous sample is taken, and again after 2 h. The samples taken are immediately processed to avoid preanalytic glycolysis. Glucose analysis is done using the enzymatic reference method with hexokinase (Hexokinase G6PDH/UV Roche Cobas^®^ c500/c303, CA, USA).

This study assessed four GDM diagnostic criteria, namely, the IADPSG, NICE 2015, WHO 1999, and ADIPS 1998. These criteria are among the commonly used by doctors in the UAE as highlighted by different studies (24, 25). Standard definitions of the criteria are given in **Supplementary Table S1**.

A sample size of 1,438 pregnancies will allow the detection of a true relative risk of approximately 1·73 (30) of developing large for gestational age (LGA) babies in GDM patients (using IADPSG) with 80% power and at 95% confidence level, accounting for a 20% attrition rate (Fleiss with CC).

### 2.4 Outcomes

The outcomes assessed were large for gestational age (LGA) and a composite outcome (three maternal and three newborn adverse outcomes). The LGA was defined as a newborn’s birth weight above the 90th percentile for gestational age and sex. The LGA was determined using the reference from the US Centers for Disease Control and Prevention growth chart and the method described by Vidmar S. I. and colleagues in 2013 (31). The composite outcome was defined as having one or more from LGA, neonatal intensive care unit (NICU) admission, abnormal APGAR score, caesarean delivery, premature delivery, and preeclampsia. These were selected based on the evidence from a meta-analysis of adverse perinatal outcomes among more than 7.5 million pregnancies and the most commonly found in the Arab population (32, 33). See **Supplementary Material (p 2)** for the operational definition of each outcome variable.

### 2.5 Statistical analysis

We summarized continuous variables using means with standard deviations (SD) and categorical variables using frequencies with percentages (%). Logistic regressions were conducted to assess the associations between the four GDM diagnostic criteria and the adverse perinatal outcomes (LGA and composite outcome). Generalized linear models (GLM) were used to estimate the adjusted relative risks (RR) and risk differences (RD) with 95% confidence intervals for these associations. Logistic regressions were also conducted to assess the associations between OGTT (FPG, 1 h, and 2 h) readings (used as continuous variables) and the outcomes. Regression results were reported using odds ratios with 95% confidence intervals. For the OGTT readings, we calculated odds ratios per unit change (1 mmol/L) in fasting, 1-h, and 2-h plasma glucose levels.

For the multiple regressions, two models (model 1 and model 2) were used. Model 1 was constant for all outcomes and adjusted for baseline maternal characteristics, including age, gravidity, body mass index (BMI) at booking, education, employment, family history of type 2 DM, previous GDM, and study center. Model 2 adjusted for model 1 plus lifestyle factors and other factors. For LGA, model 2 included model 1 plus maternal smoking, passive smoking, physical activity, antepartum hemorrhage, and previous macrosomia. For the composite outcome, a family history of hypertension was added to this list. See **Supplementary Material (p 2)** for the operational definition of each exposure variable. Missing data in this study were handled using multiple imputations by the chained equation (MICE) method with the number of imputed datasets specified at m = 100.

Following multiple regression with the OGTT results, post-estimation analysis was conducted and expressed using the *marginsplot* to show the predictive margins for the fitted, adjusted model, which was then used to make predictions. The method adopted by the UCLA Statistical Consulting Group (34), which utilizes multiple imputed data, was used. New cutoff values were identified using the adjusted predictions. New GDM diagnostic criteria was proposed for this population based on these cutoffs.

The new criteria was compared with two others (IADPSG and NICE 2015) in terms of GDM cumulative incidence, adjusted odds ratio (aOR) for outcomes with 95% confidence intervals, and agreements using kappa statistics. These two criteria were selected because they are commonly used in the UAE and were also found to be the most inclusive in GDM diagnosis for this population (3).

Further evaluation of the new criteria and the IADPSG was done using:

- The c-statistic (using area under the curve (AUC) for receiver operating characteristics (ROC)).
- The net reclassification improvement/index (NRI).
- The integrated discrimination improvement (IDI).

The AUC assessed the performance and discrimination of the IADPSG and the new criteria in two separate models containing established risk factors (models 1 and 2) of the outcomes. Results were presented as a graph using the ROC curves and showing the AUC with 95% confidence intervals for both models. AUC values of 0·9–1·0 show that tests have excellent quality; 0·8–0·9 very good quality; 0·7–0·8 good (acceptable) quality; 0·6–0·7 satisfactory; 0·5–0·6 unsatisfactory. The test of equality for the two models was conducted using the chi-square test. P-value was significant at <0·05.

The continuous NRI was used to assess the clinical utility of the new criteria by assessing the incremental value in its risk predictions of the outcomes. Results were presented as the proportions of reclassified cases and non-cases based on risk predictions with their NRI values. The IDI showed the mean difference in discrimination slopes of the two models (extended and traditional) for both criteria. Results were reported as Absolute IDI (standard errors—SE) and Relative IDI (%) (35).

All analyses were conducted using STATA statistical software version 16·1 (StataCorp LLC, College Station, TX, USA).

## 3 Results

A total of 5,295 participants were recruited in the Mutaba’ah Study from May 2017 to March 2021. Those with multiple pregnancies (323) and those with pending OGTT at the time of extraction (2,386) were excluded. Of the remaining 2,586 participants, 1 known and 39 newly diagnosed patients with diabetes were excluded. There were 97 participants with less than two readings who were also excluded. Hence, 2,449 participants were followed up in this study (**Supplementary Figure S1**).

### 3.1 Maternal characteristics

The mean maternal age of participants at booking was 30.4 ± 6.0 years, and the mean booking body mass index (BMI) was 27.7 ± 5.6 kg/m^2^. 53.4% of the participants had above high school education, and 30.7% were employed. Their mean fasting plasma glucose (FPG) was 4.6 ± 0.4 mmol/L, 1-h OGTT was 8.0 ± 1.9 mmol/L, and 2-h OGTT was 6.5 ± 1.6 mmol/L. **Table 1** shows the descriptives for maternal characteristics.

**Table 1:** Maternal characteristics of participating Emirati women (N = 2449).

Maternal characteristics	Total participants (N)*	Frequency n (%)	Mean ± SD
Age (years)	2,447		30·4 ± 6·0
Gravidity	2,449		3·5 ± 2·1
Educational status	2,255		
High school and below		1,050 (46·6)	
Above high school		1,205 (53·4)	
Employment status	2,258		
Unemployed		1,564 (69·3)	
Employed		694 (30·7)	
Booking weight (kg)	1,270		69·6 ± 14·7
Height (m)	2,093		1·6 ± 0·1
Booking BMI (kg/m^2^)	1,267		27·7 ± 5·6
Family history of diabetes	2,449	728 (29·7)	
Family history of hypertension	2,449	562 (23·0)	
Previous GDM	1,965	411 (20·9)	
Previous macrosomia	722	28 (3·9)	
Maternal smoking	2,293	38 (1·7)	
Passive smoking	2,298	1,136 (49·4)	
Physical Activity	859	366 (42·6)	
Maternal illness			
Chronic hypertension	2,449	26 (1·1)	
Gestational hypertension	2,449	31 (1·3)	
Antepartum haemorrhage	2,449	84 (3·4)	
Oral glucose tolerance test (OGTT) results in mmol/L			
Fasting plasma glucose (FPG)	1,108		4·6 ± 0·4
1-h OGTT	1,531		8·0 ± 1·9
2-h OGTT	2,443		6·5 ± 1·6

*Total number of participants with data for a variable. BMI, body mass index; kg, kilograms; m, meters; mmHg, millimeters of mercury; mmol/L, millimoles per liter; GDM, gestational diabetes mellitus.

### 3.2 Adverse perinatal outcomes

**Table 2** summarizes the adverse perinatal outcomes among the participants. 17.4% of the participants had large for gestational age (LGA) babies, and 42.1% had the composite outcome (at least one of the six specified outcomes). The descriptives of the six specified outcomes (LGA, NICU admission, abnormal APGAR score, caesarean delivery, premature delivery, and preeclampsia) are highlighted in **Supplementary Table S2**.

**Table 2:** Perinatal outcomes among participating Emirati women (N = 2449).

Perinatal outcomes	Total participants (N)*	Frequency n (%)
Large for gestational age (LGA)	2400	417 (17·4)
Composite outcome ^†^	2449	
Yes		1,031 (42·1)
No		1,418 (57·9)

*Total number of participants with data for a variable. ^†^Composite outcome = one or more of LGA, NICU admission, abnormal APGAR score, caesarean delivery, premature delivery, and preeclampsia.

### 3.3 Predictions of adverse perinatal outcomes by the four GDM diagnostic criteria

**Table 3** shows the associations between the GDM diagnostic criteria (IADPSG, NICE 2015, WHO 1999, and ADIPS 1998) and the outcomes. The IADPSG criteria had the highest odds ratio for the LGA (aOR 1.77, 95% CI 1.36–2.29) compared with the remaining three criteria after adjusting for models 1 and 2. The IADPSG criteria also had the highest adjusted odds ratio for the composite outcome (aOR 1·49, 95% CI 1.19–1.86).

**Table 3:** Comparing four GDM diagnostic criteria regarding their associations with large for gestational age (N = 2,400) and the composite outcome (N = 2,449) in the Emirati population of the UAE.

	Crude OR (95%CI)	Model 1: AOR (95% CI)	Model 2: AOR (95% CI)
Large for gestational age (LGA) ^†^		
IADPSG			
No GDM	1.00	1.00	1.00
GDM	1.90 (1.50 – 2.40) *	1.77 (1.36 – 2.28) *	1.77 (1.36 – 2.29) *
NICE 2015			
No GDM	1.00	1.00	1.00
GDM	1.63 (1.29 – 2.08) *	1.48 (1.15 – 1.90) *	1.44 (1.11 – 1.86) *
WHO 1999			
No GDM	1.00	1.00	1.00
GDM	1.64 (1.29 – 2.08) *	1.48 (1.15 – 1.91) *	1.44 (1.11 – 1.86) *
ADIPS 1998			
No GDM	1.00	1.00	1.00
GDM	1.58 (1.24 – 2.02) *	1.45 (1.12 – 1.89) *	1.43 (1.09 – 1.86) *
	Composite outcome ^‡^		
IADPSG			
No GDM	1.00	1.00	1.00
GDM	1.61 (1.33 – 1.95) *	1.46 (1.17 – 1.81) *	1.49 (1.19 – 1.86) *
NICE 2015			
No GDM	1.00	1.00	1.00
GDM	1.48 (1.22 – 1.79) *	1.34 (1.09 – 1.65) *	1.37 (1.10 – 1.70) *
WHO 1999			
No GDM	1.00	1.00	1.00
GDM	1.50 (1.24 – 1.82) *	1.35 (1.09 – 1.67) *	1.37 (1.10 – 1.70) *
ADIPS 1998			
No GDM	1.00	1.00	1.00
GDM	1.48 (1.21 - 1·80) *	1.35 (1.32 – 1.68) *	1.39 (1.11 – 1.74) *

^†^LGA—defined as birthweight above the 90th percentile for gestational age at delivery and sex of the baby (categorized as Yes/No). For LGA, model 1 adjusted for age, gravidity, booking BMI, education, employment, family history of type 2 DM, previous GDM, and study center; model 2 adjusted for model 1 plus maternal smoking, passive smoking, physical activity, antepartum hemorrhage, and previous macrosomia. ^‡^Composite outcome—defined as one or more of LGA, NICU admission, abnormal APGAR score, caesarean delivery, premature delivery, and preeclampsia (categorized as Yes/No). For composite outcome, model 1 adjusted for age, gravidity, booking BMI, education, employment, family history of type 2 DM, previous GDM, and study center; model 2 adjusted for model 1 plus maternal smoking, passive smoking, physical activity, antepartum hemorrhage, previous macrosomia, and family history of hypertension. aOR, adjusted odds ratio; CI, confidence interval; IADPSG, International Association of Diabetes and Pregnancy Study Groups; NICE, National Institute for Health and Clinical Excellence; WHO, World Health Organization; ADIPS, Australasian Diabetes in Pregnancy Society. *P value < 0·05.

Risk analysis results are reported in **Supplementary Table S3**, and the outcomes’ adjusted relative risks (RR) and risk differences (RD) were compared for the four criteria. It confirmed the IADPSG criteria as the strongest predictor for LGA (aRR 1·55, 95% CI 1.27–1.88) and composite outcome (aRR 1.22, 95% CI 1.09–1.35). The analysis showed that the excess risk of LGA identified by the IADPSG criteria was 9%, whereas it was 6% for the other three criteria. Similarly, for the composite outcome, the RD was highest with the IADPSG criteria.

### 3.4 Associations of OGTT results (as continuous variables) with adverse perinatal outcomes

**Table 4** shows the adjusted odds ratio denoting the strength of association between the FPG, 1-h, and 2-h OGTT results and the outcomes (LGA and composite outcome). FPG had the strongest positive association with both outcomes compared with 1-h and 2-h OGTT. Following adjustments for models 1 and 2, only the association between FPG and the composite outcome remained significant (aOR 1.67, 95% CI 1.21–2.28).

**Table 4:** Comparing the associations of fasting plasma glucose, 1-hr & 2-hr OGTT with large for gestational age (N=2400) and the composite outcome (N=2449) in the Emirati population of the UAE.

OGTT results (mmol/L)	Crude OR (95% CI)	Adjusted OR (95% CI)
Large for gestational age (LGA) ^a^		
Fasting plasma glucose	1.49 (1.04 – 2.14) *	1.35 (0.92 – 1.99)
1-h OGTT	1.11 (1.04 – 1.18) *	1.07 (1.00 – 1.15)
2-h OGTT	1.11 (1.04 – 1.18) *	1.07 (1.00 – 1.15)
Composite outcome ^b^		
Fasting Plasma Glucose	1.86 (1.39 – 2.49) *	1.67 (1.21 – 2.28) *
1-hr OGTT	1.10 (1.05 – 1.15) *	1.05 (0.99 – 1.11)
2-hr OGTT	1.08 (1.03 – 1.14) *	1.05 (0.99 – 1.11)

OR, odds ratio; CI, confidence interval; OGTT, oral glucose tolerance test; mmol/L, millimoles per liter. ^a^ LGA—defined as birthweight above the 90th percentile for gestational age at delivery and sex of the baby (categorized as Yes/No). For LGA, model 1 adjusted for age, gravidity, booking BMI, education, employment, family history of type 2 DM, previous GDM, and study center; model 2 adjusted for model 1 plus maternal smoking, passive smoking, physical activity, antepartum hemorrhage, and previous macrosomia. ^b^ Composite outcome—defined as one or more of LGA, NICU admission, abnormal APGAR score, caesarean delivery, premature delivery, and preeclampsia (categorized as Yes/No). For composite outcome, model 1 adjusted for age, gravidity, booking BMI, education, employment, family history of type 2 DM, previous GDM, and study center; model 2 adjusted for model 1 plus maternal smoking, passive smoking, physical activity, antepartum hemorrhage, previous macrosomia, and family history of hypertension. * P value < 0·05.

### 3.5 Prediction plot for the adjusted association between fasting plasma glucose and the composite outcome

**Figure 1:**
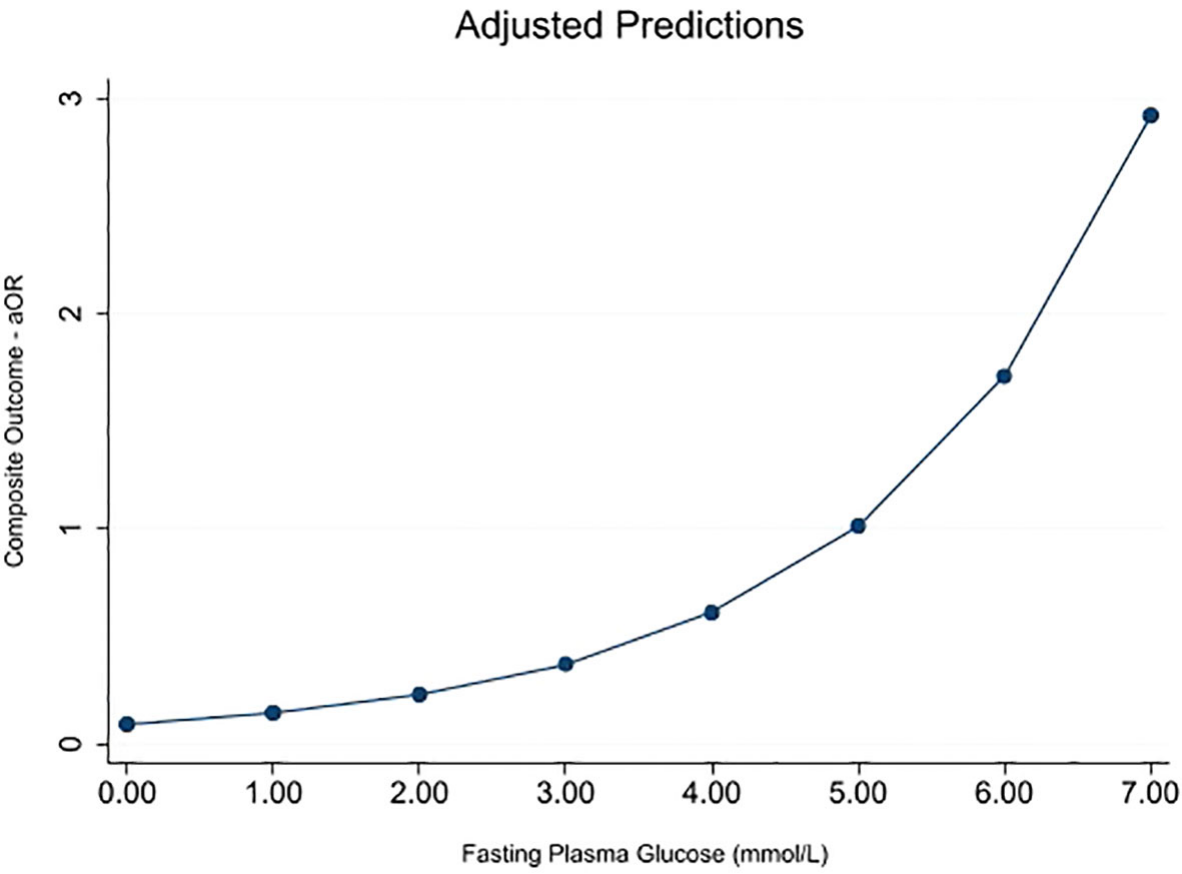
Marginsplot graphing statistics from the fitted, adjusted model (FPG vs. composite outcome) (N = 2,449). The composite outcome is defined as one or more of LGA, NICU admission, abnormal APGAR score, caesarean delivery, premature delivery, and preeclampsia. Model adjusted for age, gravidity, booking BMI, education, employment, family history of type 2 DM, previous GDM, study center, maternal smoking, passive smoking, physical activity, antepartum hemorrhage, previous macrosomia, and family history of hypertension.

Only FPG showed a significant association with the composite outcome after adjusting for models 1 and 2 (**Table 4**); hence, post-estimation was limited to its analysis. **Figure 1** shows the post-estimation prediction graphed by *marginsplot* showing the fitted, adjusted model for FPG and composite outcome. The graph shows that the association with the composite outcome starts to become positive at a maternal fasting glucose level of 5.00 mmol/L (aOR—1·01 approximately). At the recommended aOR of 1.75 (as specified by the IADPSG consensus panel), this population’s mean maternal FPG level was approximately 6.00 mmol/L.

### 3.6 Proposed new GDM criteria for the Emirati population

Based on the above prediction, we developed new GDM diagnostic criteria for the Emirati population using the FPG level of 6.0 mmol/L. For the 1-h and 2-h OGTT, thresholds of the IADPSG criteria were maintained, as it was found to be the best predictor of adverse perinatal outcomes in this population compared with other existing criteria. The new criteria was defined as maternal fasting plasma glucose (FPG) of ≥6.0 mmol/L and/or 1-hr OGTT of ≥10.0 mmol/L and/or 2-hr OGTT of ≥8·5 mmol/L following the 75-g 2-h oral glucose tolerance test.

### 3.7 Assessment of the newly proposed GDM diagnostic criteria using regression models, risk analysis, kappa statistics, and measures of performance/discrimination

#### 3.7.1 Criteria assessment (incidence, regression, risk analysis, Kappa statistics results)

**Supplementary Table S4** shows comparisons between the newly proposed criteria and two existing criteria (IADPSG and NICE) regarding their GDM incidences and adjusted odds ratios of the outcomes. The new criteria gave lower cumulative incidence (GDM Incidence—18.1%, 95% CI 16.6—19.7) than the IADPSG (21.3, 95% CI 19.8–23.0) and NICE criteria (21.5, 95% CI 19.9–23.1). However, it was a better predictor of LGA (aOR 1·93, 95% CI 1.48–2.53; aRR 1·65, 95% CI 1.35–2.01) and composite outcome (aOR 1·62, 95% CI 1.28–2.05; aRR 1.26, 95% CI 1.12–1.40) compared with the two criteria. The risk difference for both outcomes by the new criteria was 11% (**Supplementary Table S3**).

There was a strong agreement between the new criteria and the IADPSG criteria (k = 0·89; p<0·001) and a moderate agreement with the NICE 2015 criteria (k = 0·71; p<0·001) (**Supplementary Table S5**).

#### 3.7.2 Area under the ROC curve

The new criteria and the IADPSG predictive models were found to have good predictive power for both outcomes. For LGA (**Supplementary Figure S2**), the IADPSG model showed an AUC of 0.759 (95% CI; 0.661–0.858) and the new model showed an AUC of 0.776 (95% CI; 0.675–0.875), (p=0·534). For the composite outcome (**Supplementary Figure S3**), the IADPSG (AUC 0.729, 95% CI; 0.567–0.833) and the new criteria (AUC 0.730, 95% CI; 0.572–0.837) models had very similar predictive power (p=0·879).

#### 3.7.3 Net reclassification improvement

The net reclassification improvement (from the traditional model) for LGA prediction was better and more significant with the new criteria [NRI; 0·494 (p=0·043)] than with the IADPSG criteria [NRI; 0.202 (p=0·409)]. The new criteria reclassified more cases (47.4%) upward and correctly reclassified up to 76.3% of non-cases downward (**Supplementary Table S6**). The NRI for both the IADPSG and new criteria in the composite outcome prediction were not statistically significant [NRI; 0.312 (p=0·088) vs. NRI; 0.162 (p=0·376), respectively] (**Supplementary Table S7**).

#### 3.7.4 Integrated discrimination improvement

The IDI assessment showed that the new criteria model increased the discrimination slope (from the traditional model) by 42.2% for LGA and 5.0% for the composite outcome. This was much higher than the IADPSG criteria in both instances (9.0% for LGA and 1.3% for composite outcome) (**Supplementary Table S8**).

## 4 Discussion

From this representative sample of the Emirati population of the United Arab Emirates (UAE), findings show that of the four commonly used GDM diagnostic criteria in this population (25); the IADPSG criterion was the best predictor of adverse perinatal outcomes. This finding was statistically and clinically significant and corroborates the findings of other studies in the Arabian Gulf region (21–23). Further assessment showed that after adjusting for potential confounders, the maternal fasting plasma glucose in this population better predicted adverse perinatal outcomes than the 1-h and 2-h OGTT results. From post-estimation analysis, a new GDM diagnostic criteria was developed using the prediction of adverse perinatal outcomes, giving rise to a new cutoff value for the Emirati population. Since the HAPO study (14), research proposing new GDM criteria have been conducted but not based on predicting adverse perinatal outcomes (36, 37). The authors of this study propose to term this newly proposed criteria as “UAE-modified-IADPSG”. Although it is stricter in diagnosing GDM than the currently recommended IADPSG criteria, the new criterion was found to be a stronger predictor of adverse perinatal outcomes among Emirati GDM patients and was more clinically relevant with better performance and discrimination properties.

Large for gestational age (LGA) was chosen as the primary outcome because it is a direct effect of hyperglycemia in pregnancy (38). In fully adjusted regression models, out of the four GDM diagnostic criteria assessed in this study, the IADPSG criterion was found to be the strongest predictor of LGA. The NICE 2015, WHO 1999, and ADIPS 1998 predict LGA almost equally. A study in Canada showed similar results when comparing the IADPSG criteria to their national criteria (39). On the other hand, a meta-analysis of studies conducted in the US, Australia, Asia, and Europe showed that the predictions of LGA using different GDM criteria, including IADPSG, were not significant (40). Our predictions could not be compared with other studies in the Gulf region as the odds/risk ratios of perinatal outcomes were not assessed in these studies (21–23).

This study showed that of the three OGTT results, the fasting plasma glucose (FPG) was the strongest predictor for both LGA and composite outcome. However, the association between FPG and LGA was no longer significant after adjusting for the risk models. This result is consistent with the findings of a study in China (41). Using FPG alone instead of the widely accepted first-line OGTT for GDM diagnosis has been explored and recommended in some settings (42).

In 1997, at the ADA-sponsored 4th GDM International Workshop conference (43), a consensus was made to base the development of GDM diagnostic criteria on the risk of adverse perinatal outcomes, hence the Hyperglycemia and Adverse Pregnancy Outcomes (HAPO) study in 2008 (14). One of the strengths of the HAPO study included being a multicenter, multiethnic study with more than 25,000 participants. 48% of the participants were white, 12% were black, 8% were Hispanic, 29% were either Asian or Oriental, and 3% were unknown (44). Arab populations were not adequately represented in the sample. With the growing burden of GDM among the Arab population (2), the importance of accurately diagnosing the condition cannot be overemphasized.

In this study, we developed an ideal GDM diagnostic criteria for the Emirati population using the recommendations of the IADPSG consensus panel (44). Only the FPG threshold was redefined because it was the only parameter that remained significantly associated with the outcomes following adjustments in multiple regressions. This result is consistent with the findings of studies in Asia (41, 45). The pathophysiology of raised FPG and the other two OGTT results differ. Increased FPG levels have been linked to higher baseline insulin resistance compared with the 1-h and 2-h OGTT levels (46). This could explain our results among the Emirati population, which is known to have a high burden of insulin resistance and its complications (47).

Currently, the IADPSG is the locally recommended GDM diagnostic criteria in the UAE, although there is evidence that different doctors in the country use different criteria (25). Our study reiterates the relevance of the IADPSG criteria over other existing criteria in the UAE. It also revealed new evidence of a more optimal criteria than the IADPSG. Following risk analyses, the new criteria identified approximately 50 more women from our sample at risk of GDM adverse perinatal outcomes than the IADPSG did. Moreover, at least 100 more than the other criteria. Considering the fertility rate in our population (48), the new tool could identify approximately 1,000 more Emirati women at risk annually than the IADPSG. The new criteria and the IADPSG both had good and acceptable predictive power, with the AUCs of the new criteria models slightly larger than those of the IADPSG. Our findings are similar to the study on multiethnic Australian population, where the AUC of the IADPSG model was also found to be satisfactory (AUC—0.68) (11). One of the limitations of AUC is its inability to capture the clinical utility of a diagnostic tool; hence, we employed NRI and IDI for this assessment (35).

The Net Reclassification Improvement (NRI) is an index that quantifies how well a new model reclassifies cases and non-cases, correctly or incorrectly, based on the risk of outcomes compared with a baseline (traditional) risk model. It gives the proportion of cases or non-cases reclassified upward, i.e., to increased risk and vice versa for downward reclassification (35). The traditional model used in this study was a predictive model consisting of established risk factors (models 1 and 2). Our study highlighted that none of the IADPSG models (for LGA and composite outcome) significantly improved over the traditional model. The new criteria, however, significantly improved risk reclassification from the traditional model for LGA by 49.4%. This means that approximately half of the patients whose risk status changed were reclassified correctly by the new criteria. This was not significant for the composite outcome. The Integrated Discrimination Improvement (IDI) takes this one step further because it shows us the magnitude of the discrimination slopes, compared between the traditional risk model and both criteria (35). The IDI reinforced the clinical relevance of the new criteria by showing that the new criteria can predict patients with a high risk of adverse perinatal outcomes (LGA) better than the traditional model by 42.2%, whereas it is only by 9.0% for the IADPSG (compared with the same traditional model). This relevance is also reflected in the IDI for the composite outcome (new criteria by 5.0% vs. IADPSG by 1.3%).

The implications of these results are 3-fold. Firstly, our study confirms the superiority of the IADPSG criteria over existing criteria in the UAE regarding adverse perinatal outcomes risk prediction. This gives the evidence-based backing to unify doctors’ practice in the country. Secondly, this study has proved the newly proposed criteria to be a valid tool for diagnosing GDM in the Emirati population. This is the first study to develop evidence-based GDM diagnostic criteria based on adverse perinatal outcomes risk in the Arabian Gulf region. The new criteria was found to be a more optimal GDM diagnostic tool for this population than even the locally recommended IADPSG regarding risk prediction of adverse perinatal outcomes. Adopting the new criteria could lead to more targeted and effective management of GDM and its complications than the current practice by avoiding under- and overdiagnosis. Further validation of this tool is needed in reducing future type 2 diabetes risk. External validation and comparison with international data are also needed. Finally, the fact that the newly developed criteria was clinically more ideal than the IADPSG criteria in this population suggests that the worldwide unification of GDM diagnostic criteria might be challenging due to differences in the risks of GDM adverse outcomes in different populations. Hence, we recommend that experts focus on developing the optimal guidelines for unique populations, preferably at regional or national levels, to reduce the disease burden effectively (19). Strategies for translation into practice should include clinical practice evaluation, GDM guidelines and policies, education, research and development, and advocacy (**Supplementary Figure S4**).

### 4.1 Strengths and limitations

Our study’s strengths include being multicentered and conducted in a large Emirati population of the UAE, thereby increasing its generalizability and power. Rigorous methodological approaches were employed to ensure good internal validity. Regression models addressed potential predictors while keeping the bias/variance issue in mind. Finally, risk and prediction analyses provided a more relevant result for translation into clinical practice.

Limitations in this study include using non-probability (consecutive) sampling, which might affect the representativeness of our sample. Nevertheless, this issue was mitigated by the multicentered nature of our study. We did not assess all adverse perinatal outcomes due to the unavailability of the data. However, we included the primary GDM adverse perinatal outcomes expected in this population (49). The use of medical records for some variables provided incomplete data; however, we conducted a missing data analysis as described in the methods section.

### 4.2 Conclusion

Firstly, our study highlighted that the IADPSG was the best predictor of adverse perinatal outcomes out of the four commonly used GDM diagnostic criterion (IADPSG, NICE 2015, WHO 1999, and ADIPS 1998) in the UAE. Secondly, from the study data, new evidence-based GDM diagnostic criteria was developed based on the risk of adverse perinatal outcomes, which we found to be a more optimal diagnostic tool in the Emirati population than the other criteria. The new criteria could improve GDM care and reduce the burden of its perinatal complications better than the current clinical practice in this population. Following clinical trials and cost-effectiveness studies in multiethnic settings, the new criteria could be adopted widely. A multi-sectoral approach is needed to ensure the translation of this research into practice.

## Data Availability

The raw data supporting the conclusions of this article will be made available on request from The Mutaba’ah Study. Approval from the research ethics committee may be required.
