# Supplementary material for: Optimizing gestational diabetes diagnostic criteria to predict adverse perinatal outcomes in the United Arab Emirates: The Mutaba’ah Study
Source: Front Endocrinol (Lausanne). 2025 Oct 10;16:1641326. doi: 10.3389/fendo.2025.1641326 (PMC12549306; doi:10.3389/fendo.2025.1641326)

Supplementary Material

Supplemental Table S1. Definitions of the assessed GDM diagnostic criteria.

	Screened Population	Screening timing	Screening test	Abnormal values used for diagnosis	FPG (mmol/L)	1-hr OGTT (mmol/L)	2-hr OGTT (mmol/L)	3-hr OGTT (mmol/L)
IADPSG/ WHO 2013	Universal screening	24-28wks	One-Step, 2 h, 75 g	≥ 1	5·1	10·0	8·5	-
NICE 2015	Selective/ Universal screening	24-28wks	One-Step, 2 h, 75 g	≥ 1	5·6	-	7·8	-
WHO 1999 / NICE 2008	Universal screening	24-28wks	One-Step, 2 h, 75 g	≥ 1	7·0	-	7·8	-
ADIPS 1998	Universal screening	24-28wks	One-Step, 2 h, 75 g	≥ 1	5·5	-	8·0	-

FPG - Fasting blood glucose, IADPSG - International Association of Diabetes and Pregnancy Study Groups, NICE - National Institute for Health and Clinical Excellence, WHO - World Health Organization, ADIPS - Australasian Diabetes in Pregnancy Society

OPERATIONAL DEFINITIONS OF VARIABLES

Exposures

- **Age:** maternal age at pregnancy booking
- **Gravidity:** total number of pregnancies (Questionnaire)
- **Body Mass Index (BMI) at booking:** defined as weight (at booking) measured in kg divided by height measured in m^2 expressed in kg/m^2
- **Family history of DM:** cohort was divided into those who have a first degree relative with type 2 diabetes mellitus and those who do not (Yes vs No) (Questionnaire)
- **Previous GDM:** cohort was divided into those who had previous GDM and those who did not (Yes vs No) (Questionnaire)
- **Previous macrosomic baby:** cohort was divided into those who had previous macrosomic baby (≥ 4.0kg) and those who did not (Yes vs No) (Questionnaire)
- **Maternal smoking history:** cohort was divided into those who ever smoked and those who never smoked. Smoking definition includes but is not limited to the smoking of cigarette, Shisha, Midwakh etc. (Questionnaire)
- **Passive smoking:** cohort was divided into those whose husband smoke and/or are exposed to smoking at home or workplace during current pregnancy and those without smoking exposures. Smoking definition includes but is not limited to the smoking of cigarette, Shisha, Midwakh etc. (Questionnaire)
- **Physical activity**: cohort was divided into those who do physical activity at least once a week and those who do none (Yes vs No) (Questionnaire)
- **Study centre:** this refers to the recruitment hospitals (public:Al Ain and Tawam hospitals & Private: Kanad Hospital)

Outcomes

Maternal outcomes:

- **Caesarean Delivery** was defined as those who had Caesarean Section during index delivery.
- **Premature delivery** was defined as delivery before 37 completed weeks of gestation.
- **Preeclampsia** was defined using diagnosis with ICD10 codes by obstetricians.

Newborn outcomes:

- **Large for Gestational Age (LGA)** was defined as birth weight above the 90th percentile for gestational age and sex.
- **Abnormal APGAR score** was defined as 1min Apgar score of < 4 and/or 5min Apgar score of < 7.
- **NICU admission** was defined as any neonatal intensive care unit admission of a newborn lasting more than 24hrs. Admissions for newborn observation were excluded.

Supplemental Figure S1. Study flow diagram.

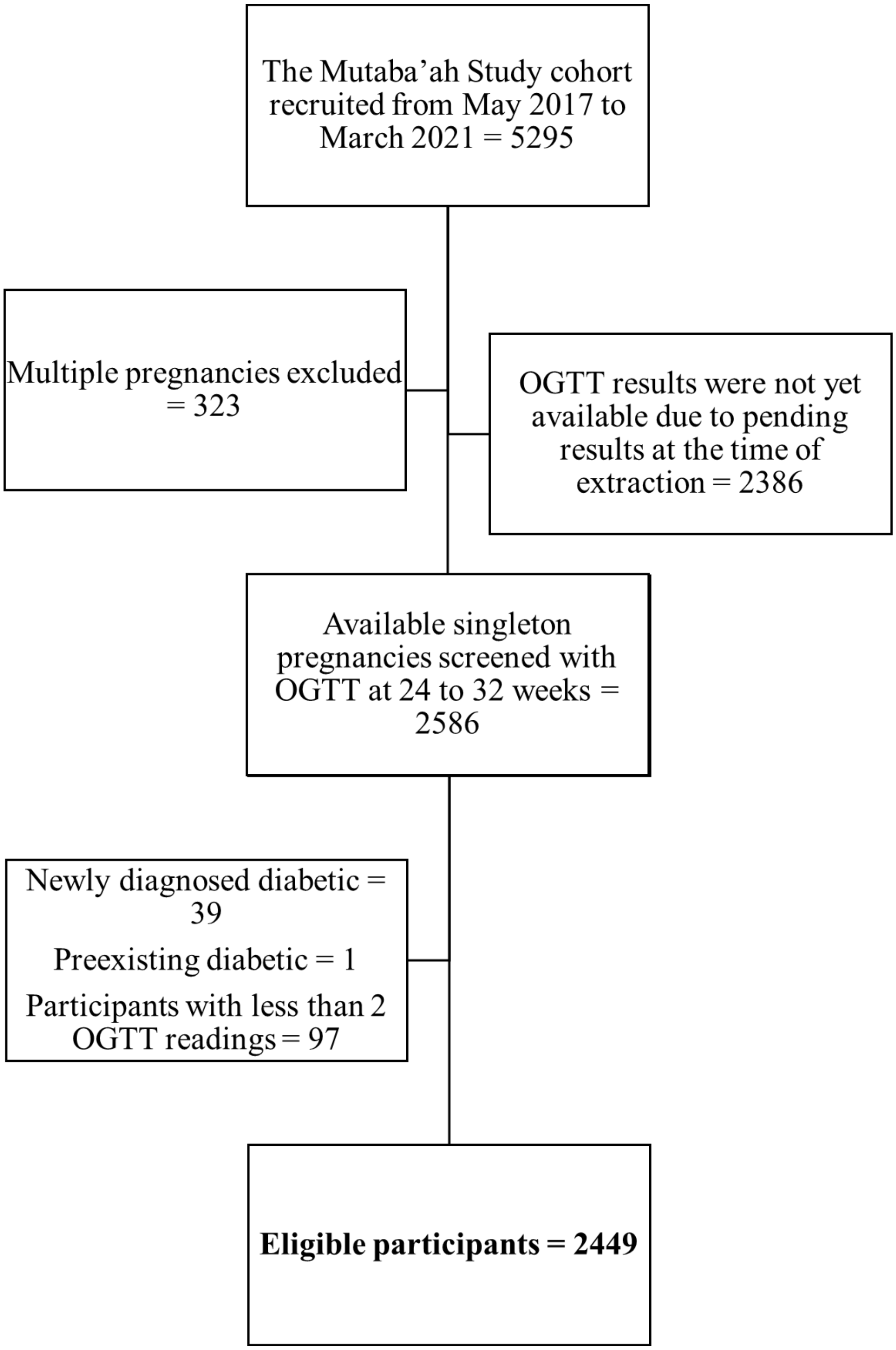

Supplemental Table S2. Adverse Perinatal Outcomes among participants (N = 2449).

New-born Outcomes	Total Participants (N) [a]	Frequency n (%)	Mean ± SD
Birthweight (g)	2406		3059.7 ± 494.0
Large for Gestational Age	2400	417 (17.4)	
Macrosomia (>4.0kg)	2406	40 (1.7)	
NICU Admission	2390	293 (12.3)	
Abnormal Apgar Score	2449	100 (4.1)	
**Maternal Outcomes**			
Caesarean Delivery	2376	614 (25.8)	
GA at Delivery (weeks)	2430		38.7 ± 1.9
Premature Delivery	2430	245 (10.1)	
Preeclampsia	2449	46 (1.9)	
**Composite Outcome** [b]	2449		
Yes		1031 (42.1)	
No		1418 (57.9)	

GA- gestational age, g – grams, kg – kilograms, NICU – neonatal intensive care unit. [a] Total number of participants with data for a variable. [b] Composite outcome = one or more of LGA, NICU admission, abnormal APGAR, Caesarean delivery, Premature delivery, & Preeclampsia.

Supplemental Table S3. Comparing the adjusted relative risks (RR) and risk differences (RD) in the associations between the assessed GDM diagnostic criteria and Large for Gestational Age (LGA) (N=2400) and the Composite Outcome (N=2449) in the Emirati population of UAE.

	Adjusted Relative Risk (95% CI)	Adjusted Risk Difference (95% CI)
	**Large for Gestational Age (LGA) [a]**	
**IADPSG**		
No GDM	1·00	
GDM	1·55 (1·27 – 1·88) *	0·09 (0·05 - 0·13) *
**NICE 2015**		
No GDM	1·00	
GDM	1·33 (1·09 – 1·61) *	0·06 (0·02 - 0·10) *
**WHO 1999**		
No GDM	1·00	
GDM	1·32 (1·09 – 1·61) *	0·06 (0·02 - 0·10) *
**ADIPS 1998**		
No GDM	1·00	
GDM	1·32 (1·08 – 1·61) *	0·06 (0·02 - 0·10) *
**New Criteria**		
No GDM	1·00	
GDM	1·65 (1·35 - 2·01) *	0·11 (0·07 - 0·15) *
	**Composite Outcome [b]**	
**IADPSG**		
No GDM	1·00	
GDM	1·22 (1·09 - 1·35) *	0·09 (0·04 - 0·14) *
**NICE 2015**		
No GDM	1·00	
GDM	1·18 (1·06 - 1·31) *	0·07 (0·02 - 0·12) *
**WHO 1999**		
No GDM	1·00	
GDM	1·18 (1·06 - 1·31) *	0·07 (0·02 - 0·12) *
**ADIPS 1998**		
No GDM	1·00	
GDM	1·19 (1·06 - 1·32) *	0·08 (0·02 - 0·13) *
**New Criteria**		
No GDM	1·00	
GDM	1·26 (1·12 - 1·40) *	0·11 (0·06 - 0·17) *

CI – confidence interval, IADPSG - International Association of Diabetes and Pregnancy Study Groups, NICE - National Institute for Health and Clinical Excellence, WHO - World Health Organization, ADIPS - Australasian Diabetes in Pregnancy Society. [a] LGA - Defined as birthweight above the 90th percentile for gestational age at delivery and sex of the baby (categorized as Yes/No). LGA was adjusted for age, gravidity, booking BMI, education, employment, family history of type 2 DM, previous GDM, study centre, maternal smoking, passive smoking, physical activity, antepartum haemorrhage, and previous macrosomia. [b] Composite outcome - Defined as one or more of LGA, NICU admission, abnormal APGAR, Caesarean delivery, Premature delivery, and preeclampsia (categorized as Yes/No). Composite outcome was adjusted for age, gravidity, booking BMI, education, employment, family history of type 2 DM, previous GDM, study centre, maternal smoking, passive smoking, physical activity, antepartum haemorrhage, previous macrosomia, and family history of hypertension. * P value < 0·05.

Supplemental Table S4. Comparing the GDM incidences and predictions of LGA and the Composite Outcome between the newly proposed criteria, IADPSG, and NICE 2015.

**GDM Criteria**	**GDM incidence** % (95% CI)	**LGA** (aOR 95% CI) [a]	**Composite Outcome** (aOR 95% CI) [b]
**New Criteria ***	18·1 (16·6 - 19·7)	1·93 (1·48 - 2·53)	1·62 (1·28 - 2·05)
**IADPSG**	21·3 (19·8 - 23·0)	1·77 (1·36 - 2·29)	1·49 (1·19 - 1·86)
**NICE 2015**	21·5 (19·9 - 23·1)	1·44 (1·11 - 1·86)	1·37 (1·10 - 1·70)

aOR – adjusted odds ratio, CI – confidence interval. * Developed with FPG level at aOR of 1.75 (of composite outcome). IADPSG - International Association of Diabetes and Pregnancy Study Groups, NICE - National Institute for Health and Clinical Excellence. [a] Defined as birthweight above 90th percentile for gestational age at delivery and sex of baby (categorized as Yes/No). [b] Defined as one or more of LGA, NICU admission, abnormal APGAR, Caesarean delivery, Premature delivery, & Preeclampsia (categorized as Yes/No). Adjusted for age, gravidity, booking BMI, education, employment, family history of type 2 DM, previous GDM, study centre, maternal smoking, passive smoking, physical activity, family history of hypertension, antepartum haemorrhage, and previous macrosomia (models 1 & 2).

Supplemental Table S5. Using kappa statistics to show agreements between the new and existing criteria.

	New criteria	IADPSG	NICE 2015
New criteria	**1·0**		
IADPSG	0·89	**1·0**	
NICE 2015	0·71	0·67	**1·0**

Cohen's Kappa Coefficient (k) interpretation; 0 - 0·20 = None, 0·21 - 0·39 = Minimal, 0·40 - 0·59 = Weak, 0·60 - 0·79 = Moderate, 0·80 - 9·0 = Strong, >9·0 = Almost perfect /Perfect. P values were <0·001 for all the comparisons (k statistics). IADPSG - International Association of Diabetes and Pregnancy Study Groups, NICE - National Institute for Health and Clinical Excellence.

Supplemental Figure S2. Graph comparing the AUC of the IADPSG and new criteria models for large for gestational age (LGA) prediction (p=0·534).

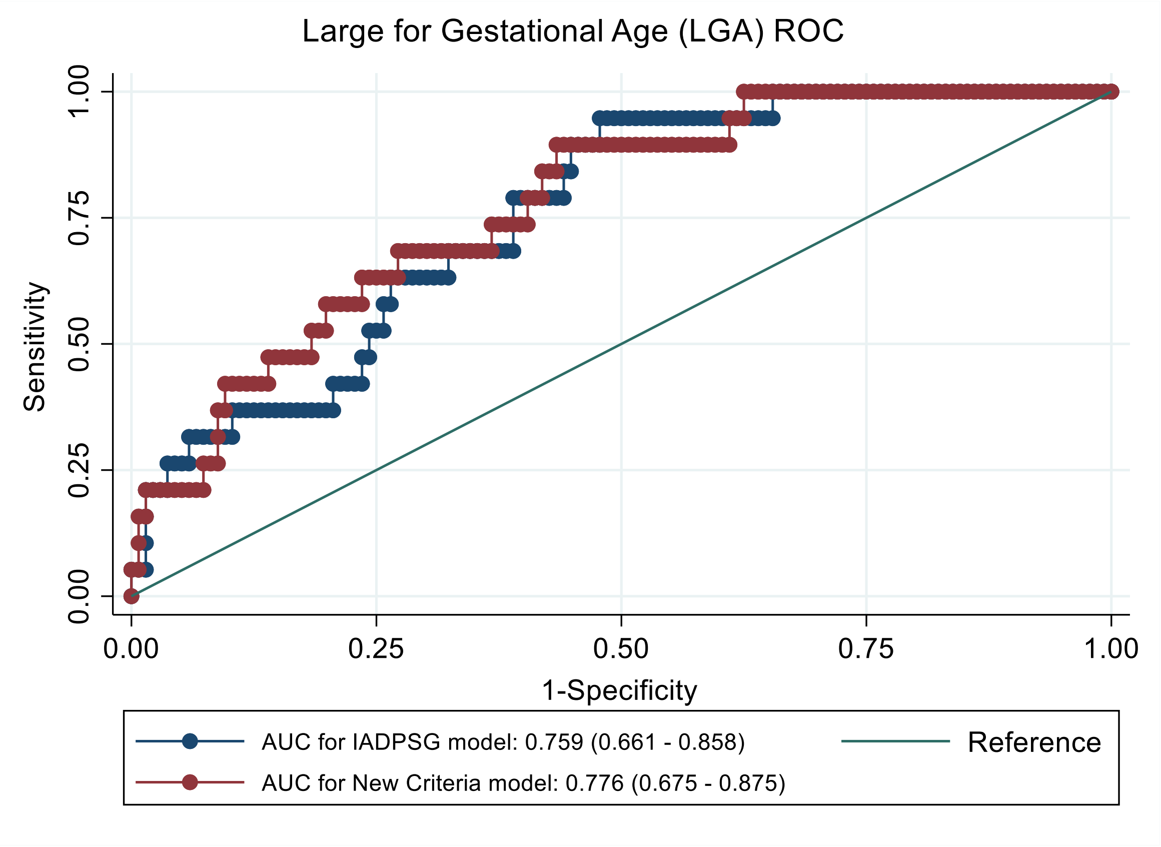

AUC - Area under the curve, ROC - Receiver Operating Characteristics, IADPSG - International Association of Diabetes and Pregnancy Study Groups.

Supplemental Figure S3. Graph comparing the AUC of IADPSG and new criteria models for Composite Outcome prediction (p=0·879).

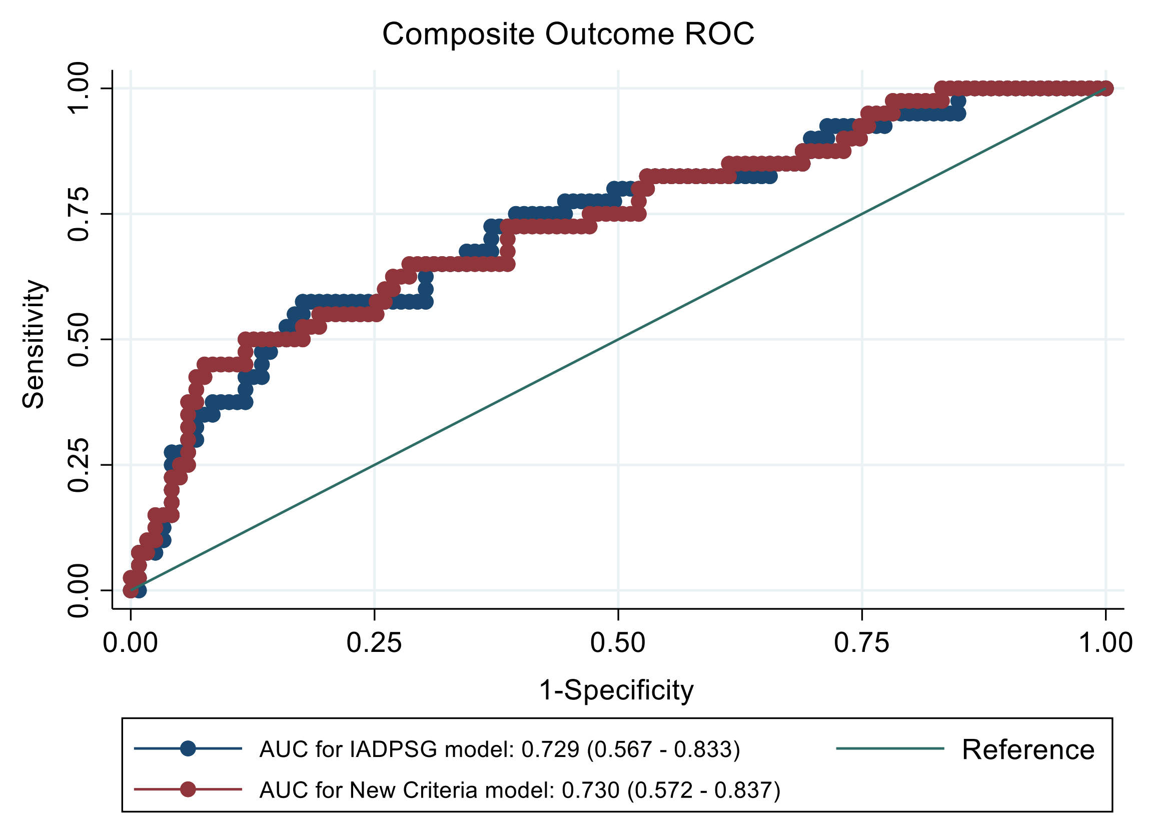

AUC - Area under the curve, ROC - Receiver Operating Characteristics, IADPSG - International Association of Diabetes and Pregnancy Study Groups.

Supplemental Table S6. Comparing the net reclassification improvements (NRI) of the IADPSG and new criteria predictive models for LGA (over traditional model).

	**IADPSG Criteria Model**			**New Criteria Model**		
**LGA**	Proportion reclassified upward (%)	Proportion reclassified downward (%)	NRI for IADPSG (p value)	Proportion reclassified upward (%)	Proportion reclassified downward (%)	NRI for New Criteria (p value)
Cases	42·1	57·9	0·202 (0·409)	47·4	52·6	0·494 (0·043)
Non-cases	30·9	66·9		21·3	76·3	

NRI – net reclassification improvement. Traditional prediction model used included age, gravidity, booking BMI, education, employment, family history of type 2 DM, previous GDM and study centre, maternal smoking, passive smoking, physical activity, antepartum haemorrhage, and previous macrosomia (Models 1 & 2).

Supplemental Table S7. Comparing the net reclassification improvements (NRI) of the IADPSG and new criteria predictive models for Composite Outcome (over traditional model).

	IADPSG Criteria Model			New Criteria Model		
**Composite Outcome**	Proportion reclassified upward (%)	Proportion reclassified downward (%)	NRI for IADPSG (p value)	Proportion reclassified upward (%)	Proportion reclassified downward (%)	NRI for New Criteria (p value)
Cases	45·0	55·0	0·312 (0·088)	37·5	62·5	0·162 (0·376)
Non-cases	29·4	70·6		29·4	70·6	

NRI – net reclassification improvement. Traditional prediction model used included age, gravidity, booking BMI, education, employment, family history of type 2 DM, previous GDM and study centre, maternal smoking, passive smoking, physical activity, antepartum haemorrhage, previous macrosomia, and family history of hypertension (Models 1 & 2).

Supplemental Table S8. Comparing the integrated discrimination improvements (IDI) of the IADPSG and new criteria predictive models (over traditional models).

	LGA		Composite Outcome	
	**IADPSG criteria**	**New Criteria**	**IADPSG criteria**	**New Criteria**
**Absolute IDI (Standard error)**	0·008 (0·010)	0·039 (0·024)	0·001 (0·004)	0·005 (0·007)
**Relative IDI ***	9·0%	42·2%	1·3%	5·0%

IDI – integrated discrimination improvement. * Relative to traditional prediction model (Models 1 & 2). Traditional prediction model used included age, gravidity, booking BMI, education, employment, family history of type 2 DM, previous GDM and study centre, maternal smoking, passive smoking, physical activity, antepartum haemorrhage, previous macrosomia, and family history of hypertension

Supplemental Figure S4. Summary of our research translation strategies for recommendations to improve GDM care in the UAE.

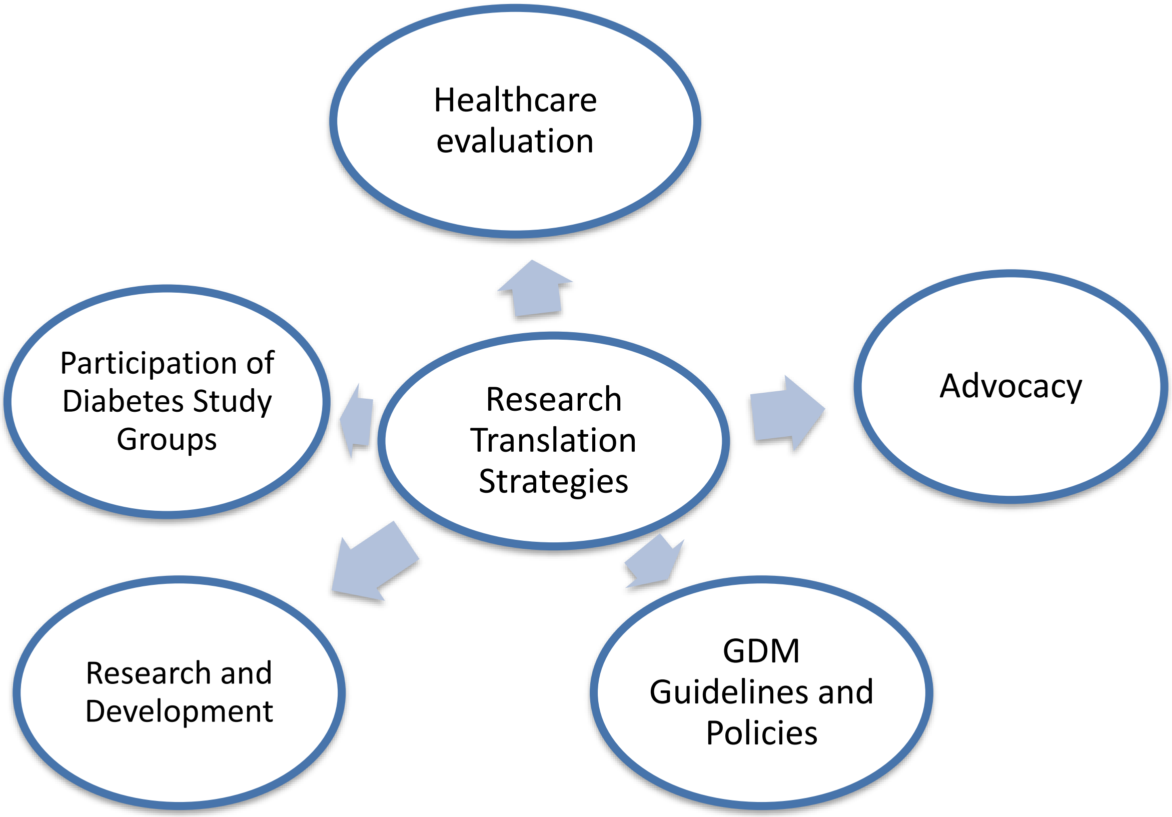

Supplement: Supplementary file 1 [file DataSheet1.pdf]
